# Cerebellar Circuits for Classical Fear Conditioning

**DOI:** 10.3389/fncel.2022.836948

**Published:** 2022-03-30

**Authors:** Kyoung-Doo Hwang, Sang Jeong Kim, Yong-Seok Lee

**Affiliations:** ^1^Department of Physiology, Seoul National University College of Medicine, Seoul, South Korea; ^2^Department of Biomedical Science, Seoul National University College of Medicine, Seoul, South Korea; ^3^Neuroscience Research Institute, Seoul National University College of Medicine, Seoul, South Korea; ^4^Wide River Institute of Immunology, Seoul National University, Hongcheon, South Korea

**Keywords:** cerebellum, fear conditioning, synaptic plasticity, microcircuits, non-motor cognitive function, emotion

## Abstract

Accumulating evidence indicates that the cerebellum is critically involved in modulating non-motor behaviors, including cognition and emotional processing. Both imaging and lesion studies strongly suggest that the cerebellum is a component of the fear memory network. Given the well-established role of the cerebellum in adaptive prediction of movement and cognition, the cerebellum is likely to be engaged in the prediction of learned threats. The cerebellum is activated by fear learning, and fear learning induces changes at multiple synaptic sites in the cerebellum. Furthermore, recent technological advances have enabled the investigation of causal relationships between intra- and extra-cerebellar circuits and fear-related behaviors such as freezing. Here, we review the literature on the mechanisms underlying the modulation of cerebellar circuits in a mammalian brain by fear conditioning at the cellular and synaptic levels to elucidate the contributions of distinct cerebellar structures to fear learning and memory. This knowledge may facilitate a deeper understanding and development of more effective treatment strategies for fear-related affective disorders including post-traumatic stress or anxiety related disorders.

## Introduction

Classical fear conditioning is widely used as a behavioral paradigm for studying fear learning and memory. Fear conditioning involves an unconditioned stimulus (US) such as an aversive footshock for inducing unconditioned responses including freezing and escaping behavior, and a conditioned stimulus (CS), which is a neutral sensory stimulus such as a salient acoustic tone that does not induce aversive responses *per se* (Myers and Davis, 2007; Tovote et al., 2015). Fear conditioning largely consists of four phases: acquisition, consolidation, retrieval, and extinction. In the acquisition phase, the CS is paired with the US to form an association of the CS and US (Myers and Davis, 2007; Tovote et al., 2015). The association of the US with either a sensory cue (CS) or with the context is termed cued or contextual fear learning, respectively (Myers and Davis, 2007; Tovote et al., 2015). This associative learning is stored as a long-term memory through the consolidation phase. In the retrieval phase, the presentation of the CS alone can induce conditioned responses such as freezing (Myers and Davis, 2007; Tovote et al., 2015). In the extinction phase, a further repetitive presentation of the CS alone decreases CS-dependent fear responses (Myers and Davis, 2007; Tovote et al., 2015). One of the main measures of fear behavior in rodents is calculated from the time spent freezing.

Although the brain regions such as the amygdala, medial prefrontal cortex, hypothalamus, hippocampus, and periaqueductal gray (PAG) have been extensively investigated to study biological mechanisms underlying fear learning and memory, accumulating evidence strongly suggests that the cerebellum also plays a critical role. The cerebellum is well known for its roles in motor control and error-based learning (Ito, 2002; Hull, 2020). In addition, it is involved in associative learning paradigms including reward learning and eyeblink conditioning by encoding sensory prediction errors and timing (Ten Brinke et al., 2017; Heffley and Hull, 2019). Considering the cerebellar functions for error-based learning and sensory prediction, the cerebellum may be one of the brain regions critically involved in fear conditioning.

**Figure 1:**
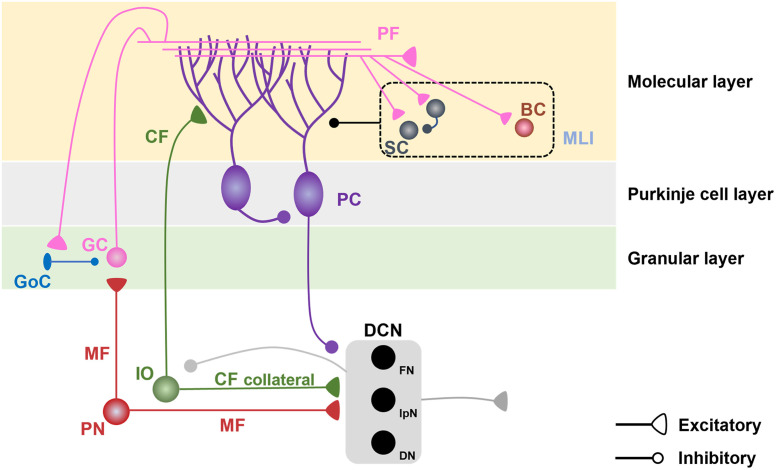
Cerebellar microcircuits. Pontine nuclei send excitatory projections to cerebellar granule cells (GCs) and deep cerebellar nuclei (DCN) *via* mossy fibers (MFs). GCs, which receive inhibitory inputs from Golgi cells (GoCs) in the granular layer, send excitatory projections to the dendrites of Purkinje cells (PCs) and molecular layer interneurons (MLIs), including stellate cells (SCs) and basket cells (BCs) in the molecular layer. MLIs send inhibitory projections to PC dendrites and have reciprocal inhibitory connections among SCs. The inferior olive (IO) sends excitatory projections to PC dendrites and DCN *via* climbing fibers (CFs) and their collaterals. PCs send inhibitory projections to the DCN and neighboring PCs. The DCN sends excitatory projections to the extracerebellar regions and inhibitory projections to the IO.

Although the cerebellum has a seemingly uniform architecture as depicted in Figure 1, it has more complex heterogeneity of cerebellar cell types and synaptic connectivity (Apps and Garwicz, 2005; Cerminara et al., 2015). The complex heterogeneous architecture of the cerebellum is underscored by several factors, including cerebellar molecular expression patterns and region-specificity (Sugihara and Shinoda, 2004; Sugihara et al., 2009; Sugihara, 2011; Fujita et al., 2020; Kebschull et al., 2020). This structural heterogeneity in the cerebellum highlights the potential involvement of the cerebellum in various motor and non-motor functions. In line with its structural heterogeneity, Purkinje cells (PCs) which are the output of the cerebellar cortex play various functional roles in the cerebellar lobule-specific locations (Apps et al., 2018). In addition, deep cerebellar nuclei (DCN) which are the sole output of the entire cerebellum and receive inhibitory projections from PCs have distinct subnuclei diversified with evolutionary processes and a variety of molecular expressions (Sugihara, 2011; Kebschull et al., 2020). Each DCN subnuclei has its own connectivity pattern and is thought to serve its own functions for learning, respectively (D’mello et al., 2020; Fujita et al., 2020; Pisano et al., 2021).

These diverse processes in each cerebellar unit of the cerebellar regions enable the cerebellum to regulate the coordination of motor and non-motor functions, including fear conditioning and social behaviors (Adamaszek et al., 2017; Badura et al., 2018; Carta et al., 2019; Jackman et al., 2020; Klaus and Schutter, 2021). Indeed, recent studies have demonstrated that fear conditioning triggers changes in plasticity at multiple loci in the cerebellum, suggesting that distinct cerebellar components are involved in fear conditioning (Strick et al., 2009; Apps and Strata, 2015; Adamaszek et al., 2017). Here, we summarize the cerebellar contributions to fear conditioning by reviewing how the cerebellum is involved in fear learning and memory at the cellular and network levels in the mammalian brain.

## The Role of The Cerebellum in Fear Learning and Memory in Humans

Studies using functional magnetic resonance imaging (fMRI) in humans have revealed cerebellar involvement in the processing of various aversive stimuli and associated learning, including fear conditioning (Ploghaus et al., 1999; Frings et al., 2002; Kattoor et al., 2014; Lange et al., 2015; Utz et al., 2015; Ernst et al., 2019; Faul et al., 2020). Ploghaus et al. (1999) examined brain regions involved in acute pain and its anticipation and reported that alongside other brain areas including the medial frontal lobe and insular cortex, the cerebellum was differentially activated by a painful thermal stimulus paired with a colored light stimulator. Although they did not specify the cerebellar regions involved in associative fear conditioning, the bilateral anterior cerebellum was predominantly activated during painful stimulation, whereas the ipsilateral posterior cerebellum was activated during anticipation of pain (Ploghaus et al., 1999). Other fMRI studies of the cerebellum in humans have investigated the neural substrates underlying associative fear learning in the cerebellum. A meta-analysis revealed that both the cerebellar vermis and hemispheres were activated in response to a CS that was paired with an US, such as an aversive electric shock (CS+), during associative fear learning in human participants (Lange et al., 2015). Another human fMRI study demonstrated that hemispheric lobule VI and the anterior vermis were strongly activated by the CS+ in the early phase of fear acquisition and early phase of extinction learning, respectively (Utz et al., 2015). Ernst et al. measured fMRI signals in the cerebellum concurrently with skin conductance responses as a proxy of fear responses during an associative fear acquisition and extinction paradigm, in which a visual stimulus (CS+) was paired with an aversive electric shock (US; Ernst et al., 2019). Significant activation was observed in lobule VI and Crus I in response to the CS+ compared to that in response to the CS− which was not paired with the US. Notably, an unexpected omission of the CS-paired US during the fear acquisition phase elicited significant activation in lobules VI and Crus I, whereas an expected US omission during the fear extinction phase did not, suggesting that the cerebellum is involved in processing aversive predictions and prediction errors (Ernst et al., 2019). Of note, significant US-elicited activation was observed predominantly in the anterior cerebellum in a study by Ploghaus et al. (1999) and in the posterolateral cerebellum, including Crus I and lobule VI, in a study by Ernst et al. (2019). This difference could be due to the different conditions employed, including the experimental settings and the manner in which human participants performed the tasks. Collectively, these converging findings highlight the involvement of the cerebellum in associative fear learning and fear extinction learning in humans, although the precise roles of distinct cerebellar regions remain to be investigated.

## The Role of The Cerebellum in Fear Learning and Memory in Rodents

Given that most human studies use imaging techniques, it is challenging to determine the causal relationship between cerebellar activation and fear learning and memory in humans. In addition, investigations of the mechanisms underlying cognitive function at the cellular level are limited in humans. In this regard, non-human animals such as rodents are widely used to examine the detailed mechanisms underlying fear learning and memory (Ledoux, 2000; Tovote et al., 2015). In this section, we review the literature on the neural substrates of associative fear learning and memory in the rodent cerebellum at the cellular level.

Cerebellar lesions or pharmacological inactivation of the cerebellar cortex or deep cerebellar nuclei have been employed to assess the contribution of the cerebellum to fear learning and memory (Supple et al., 1987, 1988; Sacchetti et al., 2002, 2007). In rats, cerebellar vermal lesions mainly targeting lobules IV and V or VIII induced a deficit in innate fear-evoked freezing to a predator (cat) with normal contextual fear memory retrieval, whereas cerebellar hemispheric lesions targeting Crus I and II induced a deficit in contextual fear memory retrieval without affecting the innate fear response to a predator (Supple et al., 1987, 1988; Koutsikou et al., 2014). It is worth noting that only contextual fear memory was assessed without the use of sensory stimuli such as a tone or light as a CS in these studies (Supple et al., 1987, 1988). Another study used a pharmacological inactivation approach with tetrodotoxin (TTX), a voltage-gated sodium channel blocker, in the cerebellar vermis or interpositus nuclei (IpN) at different post-training intervals after fear conditioning with multiple tone and foot shock pairings (Sacchetti et al., 2002). This study demonstrated that vermal inactivation induced deficits in both cued and contextual fear memory retrieval, whereas IpN inactivation induced a deficit in only cued fear memory retrieval, indicating that cerebellar activity is required for fear memory consolidation (Sacchetti et al., 2002). Notably, although the amygdala is considered a crucial site for fear memory processing, combined inactivation of the amygdala and cerebellum is required to block auditory fear memory retrieval for strong memories, suggesting that the cerebellum maybe particularly essential for processing relatively stronger fear memories (Sacchetti et al., 2007). These inactivation and lesion studies highlight the necessity of intact cerebellar activity for fear memory processing. In the following sections, we discuss the cerebellar changes induced by fear learning and memory at the synaptic and cellular levels in each cerebellar sub-region, including lobules V-VI and VIII in the cerebellar cortex, and DCN in order to shed light on the roles of the cerebellum in fear learning and memory.

### Cerebellar Cortex

In the cerebellar cortex, synaptic afferents from mossy fibers (MFs), climbing fibers (CFs), and molecular layer interneurons (MLIs) to PCs and synaptic plasticity at these synapses regulate PC firing output patterns, thereby regulating the firing of the DCN. Long-term depression (LTD) at PF-PC synapses has been suggested as the main mechanism of synaptic plasticity in cerebellar learning, including eyeblink conditioning (Ito, 2002). LTD occurs at PF-PC synapses when PFs and CFs are co-activated, mimicking the pairing of the CS and US in the associative eyeblink conditioning paradigm (Gao et al., 2012). Moreover, PF stimulation induces long-term potentiation (LTP) at PF-MLI synapses and MLI-PC synapses (Jörntell and Ekerot, 2002; Gao et al., 2012). This facilitates the inhibitory effects of the MLIs on PCs, thereby shaping the activity and regularity of PC firing. Although the eyeblink conditioning paradigm provided clues for how the cerebellum is involved in the associative learning paradigm, the fear conditioning paradigm requires non-motor components more than immediate motor reflex in the eyeblink conditioning, suggesting that fear conditioning may involve cerebellar mechanisms distinct from those in eyeblink conditioning.

Several studies have demonstrated that the cerebellar vermis is the site of convergence of the US and CS for fear-conditioned responses, including fear-evoked freezing behavior and bradycardia (Supple et al., 1987, 1988; Supple and Leaton, 1990; Sebastiani et al., 1992). Lesions in the cerebellar vermis, ranging from lobules VI to IX, induced a deficit in the acquisition of CS-dependent bradycardic responses without affecting US-dependent responses (Supple and Leaton, 1990; Sebastiani et al., 1992). At the behavioral level, lesions predominantly targeting cerebellar vermis IV and V in rats caused a deficit in a cat exposure-induced innate fear test without affecting contextual fear memory (Supple et al., 1987, 1988). Moreover, acoustic stimuli, which are typically used as the CS for associative fear learning, have been reported to converge in the cerebellar vermis (Snider and Stowell, 1944; Huang et al., 1982). These findings collectively set the basis for research on the contribution of the cerebellar vermis to fear learning and memory. In this section, we review how cerebellar microcircuits in the cerebellar vermis are involved by associative fear conditioning.

#### PF-PC Synapses

**Figure 2:**
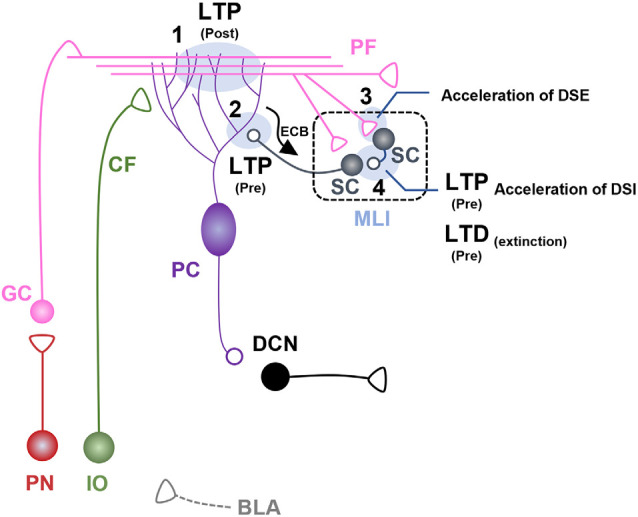
Fear learning-induced changes in cerebellar lobule V-VI microcircuits. Schematic illustration of cerebellar microcircuits regulating conditioned stimulus (CS)-dependent fear learning and memory in lobules V-VI. Each synaptic site is labeled with a number. **(1)** Postsynaptic long-term potentiation (LTP) occurs at parallel fiber (PF)-pyramidal cell (PC) synapses after auditory fear conditioning, underpinned by basolateral amygdala (BLA) activity during fear learning (Sacchetti et al., 2004; Zhu et al., 2011). **(2)** Presynaptic LTP occurs at molecular layer interneuron (MLI)-PC synapses after auditory fear conditioning (Scelfo et al., 2008; Dubois et al., 2020). PC-driven regulation of endocannabinoid signaling at MLI-PC synapses is involved in fear learning and memory (Dubois et al., 2020). **(3)** Auditory fear conditioning induces acceleration of depolarization-induced suppression of excitation (DSE) at PF-stellate cell (SC) synapses. **(4)** Auditory fear conditioning induces presynaptic LTP and accelerated depolarization-induced suppression of inhibition (DSI) at SC-SC synapses. Fear extinction learning induces presynaptic long-term depression (LTD) at SC-SC synapses (Dubois and Liu, 2021).

While LTD at PF-PC synapses is classically considered to be the neural correlate of motor learning (Ito, 2002), LTP at these synapses has been suggested to be crucial for fear learning and memory. In rats, postsynaptic LTP at PF-PC synapses in cerebellar vermal lobules V-VI was observed after auditory fear conditioning, but not after unpaired auditory fear learning (Sacchetti et al., 2004). Moreover, *hotfoot* mice that lack postsynaptic glutamate receptor delta2 at PF-PC synapses exhibited deficits in both short-term and long-term cued fear memory retrieval with intact contextual fear memory retrieval (Sacchetti et al., 2004). Genetic deletion of genes encoding *cerebellin1* in granule cells which is a ligand for postsynaptic glutamate receptor delta2 at PF-PC synapses also impaired fear acquisition, which induced deficits in both the contextual and auditory fear memory retrieval (Otsuka et al., 2016). LTP at PF-PC synapses induced by PF stimulation at 1 Hz in *ex vivo* slices was occluded 24 h after rats were fear-conditioned with a tone-shock pairing, suggesting that cued fear conditioning induced LTP at PF-PC synapse (Zhu et al., 2007). Electrically induced LTD at PF-PC synapses *via* co-stimulation of PF and CF at 1 Hz in slices was occluded at 10 min but not 24 h after electrical foot-shock stimulation, regardless of whether the foot-shocks were paired or unpaired with an auditory cue, suggesting that aversive stimuli induce LTD at PF-PC synapses immediately but not 24 h after stimulation (Zhu et al., 2007). These data strongly suggest that synaptic LTP at PF-PC synapses is critically involved in associative auditory fear conditioning (Figure 2).

Despite the key role of PF-PC synapses in lobule V-VI for fear learning and memory, the neuronal inputs contributing to synaptic regulation at PF-PC synapses remain unclear. The basolateral amygdala (BLA) is a candidate region for potentiating PF-PC synapses after auditory fear conditioning (Zhu et al., 2011). BLA inactivation *via* administration of muscimol or anisomycin immediately before or after auditory fear conditioning blocked the synaptic potentiation of PF-PC synapses, suggesting that inputs from the BLA may contribute to the modulation of PF-PC synapses in lobule V-VI (Zhu et al., 2011; Figure 2).

A recent study reported that PC-specific STAT3 knockout mice exhibited enhanced long-term auditory fear memory with normal short-term auditory fear memory and contextual fear memory (Han et al., 2021). These mutant mice demonstrated an increase in a-amino-3-hydroxy-5-methyl-4-isoxazole propionic acid (AMPA) receptor (AMPAR) expression and AMPAR-mediated currents at PF-PC synapses in lobule V-VI, whereas spontaneous gamma aminobutyric acid (GABA) release was decreased at MLI-PC synapses in lobule V-VI at the basal state compared to that in wild-type (WT) mice (Han et al., 2021). Of note, in this mouse model, an LTP induction protocol involving stimulation of PFs at 1 Hz induced LTD instead of LTP at PF-PC synapses (Han et al., 2021). Moreover, fear learning-induced LTP at PF-PC synapses was significantly reduced in PC-specific STAT3 KO mice compared to that in WT mice (Han et al., 2021). These findings support the role of PF-PC LTP in cued fear learning and memory and highlight the importance of the balance between excitatory and inhibitory synapses in the cerebellar cortex. Accordingly, synaptic plasticity at inhibitory synapses has also been reported, as discussed below.

#### MLI-PC Synapses

MLIs, including SCs and BCs, send inhibitory projections to PCs, thereby shaping the rate and pattern of PC firing (Brown et al., 2019). Auditory fear conditioning induced presynaptic LTP at MLI-PC synapses, which subsequently increased GABA-mediated inhibitory transmission to PC in lobules V-VI (Scelfo et al., 2008; Dubois et al., 2020). Auditory fear conditioning also increased spike probability with short delays in the PF-MLI-PC circuit (Scelfo et al., 2008). Moreover, endocannabinoid signaling is involved in auditory fear learning (Dubois et al., 2020). Auditory fear conditioning accelerated the endocannabinoid degradation mediated by monoacylglycerol lipase (MAGL), an enzyme that degrades endocannabinoids (Dubois et al., 2020). Chemogenetic activation of PCs reduced MAGL expression in the MLI and disrupted auditory fear memory consolidation, which was restored by blocking endocannabinoid signaling (Dubois et al., 2020). These studies suggest that MLI-PC synapses play a role in auditory fear conditioning by regulating GABA release and endocannabinoid degradation (Figure 2). A recent study, however, showed that mice with functional removal of GABA_A_-mediated signaling at MLI-PC synapses showed intact fear learning and memory (Marshall-Phelps et al., 2020), suggesting that the role of MLI-PC synapse in fear learning and memory remains to be further investigated.

#### PF-SC Synapses

PFs send excitatory projections to SCs in the molecular layer, thereby controlling the excitability and synchronization of PCs (Mittmann et al., 2005). A previous study revealed that synaptic LTD or LTP at PF-SC synapses was induced by PF stimulation at 2 Hz or pairing of PF stimulation with postsynaptic depolarization in SCs (Rancillac and Crépel, 2004). Auditory fear conditioning accelerated the recovery of depolarization-induced suppression of excitation (DSE) at PF-SC synapses, which was dependent on endocannabinoid degradation in lobule V-VI (Dubois et al., 2020). These findings imply that auditory fear conditioning changes the cerebellar microenvironment, including the DSE at PF-SC synapses, thereby regulating synaptic plasticity at PF-SC synapses (Figure 2).

Fox urine is widely used as an innate fear-evoking stimulus (Silva et al., 2016). Exposure to fox urine altered postsynaptic AMPAR complexes from GluR2-lacking AMPARs to GluR2-containing AMPARs at PF-SC synapses, which decreased calcium influx and downstream signaling activation in a beta-adrenergic receptor-dependent manner (Liu et al., 2010). Moreover, exposure to fox urine prolonged excitatory post-synaptic current (EPSC) decay time in postsynaptic SCs. The effects of fear conditioning on EPSC kinetics remain to be examined; however, the short-term kinetic difference in EPSCs may affect action potential probability in SCs, thereby regulating PC excitability (Savtchouk and Liu, 2011).

#### SC-SC Synapses

SCs are a subset of MLIs comprising GABAergic neurons that send inhibitory projections to PCs and other SCs in the molecular layer (Kondo and Marty, 1998). As mentioned above, SCs receive excitatory inputs from PFs for the feed-forward inhibition of PCs (Mittmann et al., 2005). A recent study using computational modeling demonstrated that local inhibitory circuits at SC-SC synapses in the molecular layer may regulate PC gain by affecting inhibitory circuits at MLI-PC synapses (Rizza et al., 2021). Auditory fear conditioning induced a persistent increase in GABA release at presynaptic sites of SC-SC synapses, which was reversed by fear extinction learning in lobule V-VI (Dubois and Liu, 2021). Of note, repetitive PF stimuli mimicking repetitive CS presentations for extinction learning induced a decrease in GABA release at SC-SC synapses in conditioned mice, but not in naïve mice (Dubois and Liu, 2021). GluN2D knockout mice did not exhibit PF stimuli-induced reduction in GABA release after fear conditioning (Dubois and Liu, 2021). Moreover, these mutant mice exhibited a significant deficit in fear extinction learning, suggesting that presynaptic plasticity at SC-SC synapses may be critical for fear extinction learning (Dubois and Liu, 2021). Auditory fear conditioning also accelerated the recovery of depolarization-induced suppression of inhibition (DSI) at SC-SC synapses, and this dynamic regulation was dependent on PC activation-derived endocannabinoid degradation (Dubois et al., 2020). These findings collectively suggest that SC-SC synapses are involved in the modulation of fear extinction *via* reciprocal interactions with PCs (Figure 2).

#### CF-PC Synapses

CFs from the inferior olive (IO) innervate PCs and send errors or teaching signals during motor learning (De Zeeuw and Ten Brinke, 2015; Ten Brinke et al., 2017). At the synaptic level, heterosynaptic inputs *via* CFs to PCs contribute to the formation of synaptic plasticity at PF-PC synapses by inducing widespread dendritic calcium influx, thereby inducing plasticity at PF-PC synapses (Coesmans et al., 2004). In addition to their role in encoding error signals for learning, CFs have been reported to signal reward expectation in a sensorimotor task (Kostadinov et al., 2019). CF activity has been implicated in the recognition and expectation of error signals, regardless of the valence of the errors (Ten Brinke et al., 2017; Kostadinov et al., 2019). In this regard, CF activity may also be involved in fear learning and memory. CF inputs to PCs may have distinct lobule-specific roles in fear processing. A previous study demonstrated that auditory fear conditioning did not induce any changes in the experimental parameters, including CF stimulation-induced EPSCs and paired pulse stimulation-induced depression at CF-PC synapses in lobule V-VI (Sacchetti et al., 2004). However, in lobule VIII, which will be discussed in more detail in Section “Cerebellar Cortex Lateral Vermal Lobule VIII”, inactivation of CF activity abolished ventrolateral periaqueductal gray (vlPAG) stimulation-induced increases in muscle tone, which was necessary for freezing behavior (Koutsikou et al., 2014).

#### PCs

PCs are the sole output neurons of the cerebellar cortex that send inhibitory projections to the DCN and vestibular nuclei. Their firing activity subsequently modulates the output from the DCN or VN to extra-cerebellar brain regions. As mentioned above, postsynaptic LTP was induced by auditory fear conditioning at PF-PC synapses in lobule V-VI, but intrinsic excitability and other membrane properties were not affected by fear conditioning, although the spontaneous firing of PCs was increased by fear conditioning (Zhu et al., 2006; Han et al., 2021). Moreover, enhanced cued fear memory in PC-STAT3 KO mice was not accompanied by any changes in the spontaneous firing of PCs in lobule V-VI (Han et al., 2021). Chemogenetic activation of PCs immediately after auditory fear conditioning disrupted the consolidation of long-term cued fear memory, which was shown to be mediated by PF-MLI-PC circuits (Dubois et al., 2020). This PC activation-driven disruption of cued fear memory consolidation was restored by blocking the endocannabinoid signaling pathway *via* MLI-PC synapses (Dubois et al., 2020). These findings suggest that although the intrinsic plasticity of PCs does not constitute a neural substrate for fear learning and memory, it retrogradely affects presynaptic areas, including PF terminals and MLIs, *via* the regulation of endocannabinoid signaling (Zhu et al., 2006; Dubois et al., 2020). Notably, genetically induced reduction of tyrosine hydroxylase (TH), which is a marker of catecholamines including norepinephrine (NE) and dopamine (DA), in PCs also induced a deficit in cued fear discrimination in mice (Locke et al., 2020). Although the precise roles of the catecholaminergic system in the cerebellum are unclear, it is possible that the catecholaminergic system contributes to non-motor functions, including learning of associative salient cues in the cerebellum, as in other brain regions. These TH^+^ fibers in PCs predominantly innervate the dentate nuclei (DN), which will be discussed in Section “The Role of DCN in Fear Learning and Memory in Rodents”.

### Cerebellar Cortex Lateral Vermal Lobule VIII

**Figure 3:**
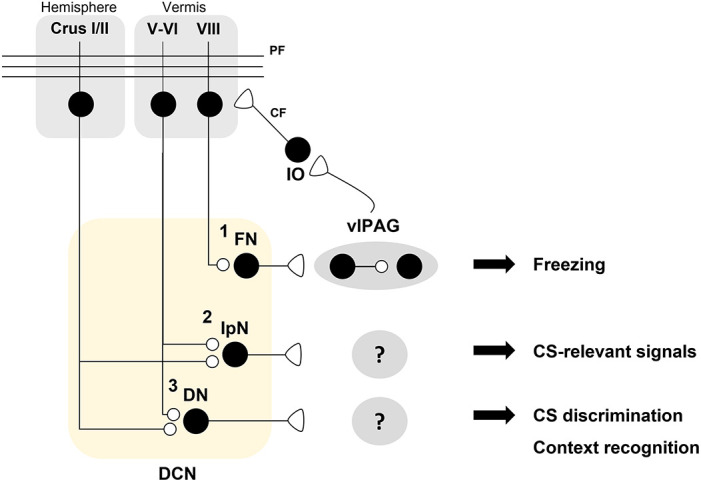
A hypothetical model for distinct roles of deep cerebellar nuclei (DCN) sub-nuclei in fear conditioning. A schematic illustration of the hypothesis that DCN sub-nuclei including the fastigial nuclei (FN), interpositus nuclei (IpN), and dentate nuclei (DN) play distinct roles in fear processing. **(1)** The FN innervates dopaminergic interneurons which regulate the freezing-regulating ChX10^+^ neurons in a D2R-dependent manner in the ventrolateral periaqueductal gray (vlPAG; Vaaga et al., 2020). Bi-directional modulation of the FN-vlPAG circuit positively regulates conditioned stimulus (CS)-dependent fear extinction learning (Frontera et al., 2020). vlPAG stimulation evokes inferior olive (IO)-mediated synaptic inputs to pyramidal cells (PCs) in lobule VIII (Koutsikou et al., 2014). Cholera toxin b (CTb)-saporin treatment in lobule VIII abolishes both vlPAG activation-induced facilitation of the H-reflex and freezing behavior in response to an innate fear-evoking stimulus and a CS (Koutsikou et al., 2014). **(2)** The IpN is necessary for the consolidation of auditory fear memory (Sacchetti et al., 2002). It is hypothesized that the IpN receives PC inputs from lobule V-VI or other hemispheric regions for encoding CS-related signals. **(3)** The DN may contribute to CS discrimination as well as contextual recognition for fear learning and memory. Crus I/II, which are required for contextual fear memory, are thought to contribute to fear memory processing in the DN (Supple et al., 1988).

While the role of lobule V-VI in fear learning and memory has been studied extensively, the contributions of other cerebellar regions in fear learning and memory remain to be investigated. Lateral vermal lobule VIII has been implicated in the regulation of motor responses in fear-induced freezing behavior (Koutsikou et al., 2014). Koutsikou and colleagues reported that electrical stimulation of the vlPAG induced a cerebellar cortical field potential, which was accompanied by complex spike activity in lobule VIII (Koutsikou et al., 2014). Electrical stimulation of the vlPAG elicited an increased amplitude of H-reflex which is an indirect but reliable readout of α-motoneuron excitability which is thought to generate muscle tone for fear-induced freezing (Koutsikou et al., 2014). Treatment with the neurotoxin tracer cholera toxin b-saporin (CTb-saporin) or *trans*-crotononitrile (TCN) into lobule VIII or the caudal IO blocked the vlPAG stimulation-induced H-reflex (Koutsikou et al., 2014). Consistent with this, CTb-saporin-mediated lesions in the lateral vermal lobule VIII induced deficits in both cued fear responses and cat odor-induced innate fear responses with increased risk assessment behavior (Koutsikou et al., 2014). These findings suggest that the vlPAG-IO-PC (lobule VIII) circuit encodes both innate and learning-dependent freezing behavior (Figure 3).

### The Role of DCN in Fear Learning and Memory in Rodents

DCN receive excitatory inputs from MFs and CF collaterals and inhibitory inputs from PCs, and project to extra-cerebellar regions as the sole output of the cerebellum (Cerminara et al., 2015; Fujita et al., 2020; Pisano et al., 2021). These DCN-centered cerebellar microcircuits have complex patterns of afferents and efferents alongside heterogeneous cerebellar molecular patterns, including zebrin and other molecular markers (Sugihara and Shinoda, 2004; Chung et al., 2009; Sugihara et al., 2009; Sugihara, 2011; Fujita et al., 2020; Henschke and Pakan, 2020; Pisano et al., 2021). The ontogenetic sequence of DCN development that elicits distinct molecular and evolutionary characteristics in each region of the DCN *via* repetitive duplication and transcriptomic divergence suggests distinct functional roles of each sub-region of the DCN in specific behavioral patterns (Kebschull et al., 2020). Although region-specific functions warrant further investigation, several studies have suggested that each DCN subregion regulates a distinct aspect of learning, including motor and non-motor components (D’mello et al., 2020; Wang et al., 2020). Molecularly or afferent-specific neuronal subgroups in the DCN have distinct projection patterns to extra-cerebellar regions (Fujita et al., 2020; Henschke and Pakan, 2020; Pisano et al., 2021). Consistent with this, fastigial nuclei (FN), interpositus nuclei (IpN), and dentate nuclei (DN), which are sub-regions classified along the mediolateral axis in the DCN, also have different efferent patterns (Kebschull et al., 2020).

Recent studies have begun to address how DCN and output projections modulate fear learning and memory (Figure 3). Neural projections from the FN to vlPAG have been implicated in the regulation of fear responses (Frontera et al., 2020; Vaaga et al., 2020). The FN sends glutamatergic projections to TH^+^ dopaminergic neurons in the vlPAG (Vaaga et al., 2020). These dopaminergic neurons negatively modulate Chx10+ neurons, which excite the magnocellular reticular nucleus of the caudal medulla to drive freezing behavior *via* D2 receptor activation (Vaaga et al., 2020). Consistent with the finding that the FN negatively regulates the freezing-inducing vlPAG circuit (Vaaga et al., 2020), bi-directional manipulations of FN-vlPAG circuit activity during fear conditioning or extinction learning have revealed that this circuit negatively regulates freezing responses (Frontera et al., 2020). Chemogenetic inhibition of the FN-vlPAG circuit during auditory fear conditioning or fear extinction learning, but not during consolidation, attenuated extinction learning (Frontera et al., 2020). Conversely, chemogenetic or optogenetic activation of the FN-vlPAG circuit during auditory fear conditioning and extinction learning, but not during consolidation, accelerated extinction learning (Frontera et al., 2020). Based on the view that fear extinction learning parallels a decrease in freezing behavior, the positive regulation of the FN-vlPAG circuit for fear extinction learning was negatively correlated with freezing behavior (Frontera et al., 2020; Vaaga et al., 2020). Considering the vlPAG is involved in generating a fear prediction error as well as pain processing, Frontera and colleagues suggest that the FN participate in the propagation of fear prediction to the vlPAG (Frontera et al., 2020).

As mentioned earlier, TTX treatment of the IpN after auditory fear conditioning suppressed the consolidation of auditory fear memory without affecting contextual fear memory, suggesting that IpN activity is required for the consolidation of cued fear memory (Sacchetti et al., 2002). Given that the IpN acts as a node for the convergence of distinct components including the US and CS in an eyeblink conditioning paradigm, it is highly likely that the IpN plays critical roles in fear memory by integrating the US and CS, although this remains to be examined (Ten Brinke et al., 2017; Wang et al., 2020).

Although direct evidence for the role of the DN in fear learning and memory is lacking, catecholaminergic input from the locus coeruleus (LC) or Purkinje cells to the DN has been reported to contribute to fear learning and memory (Locke et al., 2020; Carlson et al., 2021). In mice, genetic deletion of *Th* fibers innervating the DN, which contains the most TH^+^ fibers in the cerebellum, induced a deficit in auditory fear discrimination (Carlson et al., 2021). TH^+^ fibers in the DN predominantly originate from the LC and PCs (Carlson et al., 2021). In mice, PC-specific reduction of TH expression also disrupted auditory fear discrimination (Locke et al., 2020). Collectively, these data suggest that catecholaminergic inputs to the DN contribute to cued fear discrimination.

## Discussion

In this review, we aimed to provide a deeper understanding of the involvement and regulatory roles of the cerebellum in fear learning and memory by discussing the impact of fear conditioning on cerebellar microcircuits (Table 1). The experimental findings from classical lesion studies and recent studies involving cerebellar manipulations and cerebellar circuit mechanisms collectively suggest that the medial cerebellum, including vermal lobules V-VI, VIII, and the FN, is critically involved in the regulation of fear learning and memory. Specifically, the vlPAG, which sends IO-mediated efferent projection to lobule VIII and simultaneously receives afferents from the FN, interacts with the medial cerebellum to regulate fear learning and memory, highlighting the existence of a closed loop comprising the lobule VIII-FN-vlPAG circuit. In a viewpoint that the vlPAG is involved in generating prediction errors which is essential for fear conditioning, FN to vlPAG circuit may contribute to adjusting fear memory strength (Ozawa and Johansen, 2018; Ernst et al., 2019; Frontera et al., 2020).

**Table 1:** A summary for cerebellar involvement in fear conditioning.

Species	Region	Method	Findings	Author (years)
Human	Bilateral anterior cerebellum	fMRI	Activated by US during fear acquisition	Ploghaus et al. (1999)
	Ipsilateral posterior cerebellum	fMRI	Activated by CS during fear memory retrieval	Ploghaus et al. (1999)
	Vermis and Hemispheres	fMRI	Activated by CS during fear acquisition	Lange et al. (2015)
	Hemispheric lobule VI	fMRI	Activated by CS during fear acquisition	Utz et al. (2015)
	Anterior Vermis	fMRI	Activated by CS during fear memory extinction	Utz et al. (2015)
	Lobules VI Crus I	fMRI	Activated by CS and unexpected US omission during fear acquisition, fear memory retrieval	Ernst et al. (2019)
Rat	Vermis	Inactivation with TTX	Deficits in both cued and contextual fear memory retrieval	Sacchetti et al. (2002)
	Lobules IV-V	Lesion with tissue aspiration	Deficit in innate fear-evoked freezing to a predator	Supple et al. (1987)
	PF-PC (Lobule V-VI)	*Ex-vivo* slice recording	LTP after auditory fear acquisition	Sacchetti et al. (2004)
			LTP occlusion after auditory fear acquisition	Zhu et al. (2007)
			LTP deficit by BLA inactivation	Zhu et al. (2011)
	CF-PC (Lobule V-VI)	*Ex-vivo* slice recording	No change after auditory fear acquisition	Sacchetti et al. (2004)
	PC (Lobule V- VI)	*Ex-vivo* slice recording	No change in membrane properties after auditory fear acquisition	Zhu et al. (2006)
	MLI-PC (Lobule V-VI)	*Ex-vivo* slice recording	Increase in presynaptic GABA release after auditory fear acquisition	Scelfo et al. (2008)
	vlPAG-IO-Lobule VIII	EMG recording *in vivo* recording	vlPAG-induced muscle tone regulated by PCs in lobule VIII	Koutsikou et al. (2014)
	Vermis Amygdala	Inactivation with TTX	Required for strong fear memories	Sacchetti et al. (2007)
	Crus I and II	Lesion with electric shocks	Deficit in contextual freezing	Supple et al. (1988)
	IpN	Inactivation with TTX	Deficit in cued fear memory retrieval	Sacchetti et al. (2002)
Mouse	PF-PC	Genetic deletion of *Cerebellin1*	Deficit in fear acquisition	Otsuka et al. (2016)
		Genetic deletion of *GluRδ2* (hotfoot mice)	Deficits in both short-term and long-term cued fear memory retrieval	Sacchetti et al. (2004)
	SC-PC (Lobule V-VI)	*Ex-vivo* slice recording Immunostaining	Increase in presynaptic GABA release after auditory fear acquisition	Dubois et al. (2020)
			Accelerated ECB degradation after auditory fear acquisition
	PC	PC-specific genetic deletion of *Th*	Deficit in auditory fear discrimination	Locke et al. (2020)
	PC (Lobule V-VI)	*Ex-vivo* slice recording	LTD induced by LTP-inducing 1 Hz PF-PC stimulation	Han et al. (2021)
		Chemogenetic activation	Deficit in auditory fear memory consolidation	Dubois et al. (2020)
		PC-specific genetic deletion of *STAT3*	Enhanced long-term cued fear memory retrieval	Han et al. (2021)
	PF-SC (Lobule V-VI)	*Ex-vivo* slice recording	Altered postsynaptic AMPAR complexes by a fox urine stimulus	Liu et al. (2010)
			Increased EPSC decay time by a fox urine stimulus	Savtchouk and Liu (2011)
	SC-SC (Lobule V-VI)	*Ex-vivo* slice recording	Increase in presynaptic GABA release by auditory fear acquisition
			Recovery of presynaptic GABA release after fear extinction learning	Dubois and Liu (2021)
			Decrease in presynaptic GABA release by repetitive PF stimuli in conditioned mice
	SC (Lobule V-VI)	*Ex-vivo* slice recording	Enhanced action potential probability by a fox urine stimulus	Savtchouk and Liu (2011)
	Th+ fibers	Deletion of Th+ fibers innervating the DN	Deficit in auditory fear discrimination	Carlson et al. (2021)
	FN-vlPAG	Chemogenetic activation during acquisition/extinction	Accelerated fear extinction learning	Frontera et al. (2020)
		Chemogenetic inhibition during acquisition/extinction	Attenuated fear extinction learning
		Slice recording	Negatively regulates freezing-inducing vlPAG neurons	Vaaga et al. (2020)

*Abbreviation: AMPAR, α-amino-3-hydroxy-5-methyl-4-isoxazole propionic acid receptor; BLA, basolateral amygdala; CF, climbing fiber; CS, conditioned stimuli; ECB, endocannabinoid; FN, fastigial nuclei; fMRI, functional magnetic resonance imaging; GABA, gamma aminobutyric acid; IpN, interpositus nuclei; IO, inferior olive; LTP, long-term potentiation; LTD, long-term depression; MLI, molecular layer interneuron; PF, parallel fiber; PC, purkinje cell; SC, stellate cell; Th, tyrosine hydroxylase; TTX, tetrodotoxin; US, unconditioned stimuli; vlPAG, ventrolateral periaqueductal gray*.

Several studies have also demonstrated that the IpN and DN are involved in cued fear memory retrieval and cued discrimination, respectively, although further investigations are warranted to verify these findings (Sacchetti et al., 2002; Carlson et al., 2021). The synaptic changes induced by associative fear conditioning in lobule V-VI may contribute to neural activity in the IpN and DN underscoring fear learning and memory at the non-motor behavior level. Of note, cerebellar hemispheric regions, including Crus I and II, also contribute to fear learning and memory, as demonstrated in human and animal studies. Based on the anatomical connections and evolutionary traces of the cerebellum, it is plausible that connections from the hemispheric cerebellar cortex to the IpN and DN might be critically involved in the cognitive regulation of fear learning and memory.

One outstanding question is how the cerebellum can be integrated into the “classic” fear memory network including the amygdala. Cerebellar outputs from the DCN to other extra-cerebellar regions that modulate fear learning and memory remain to be further investigated (Figure 3). Although we have provided an extensive review of the literature on how cerebellar circuits are modified and affect fear learning and memory, the only functionally verified cerebellar output circuit is the FN-vlPAG circuit. In this regard, it remains unclear how the activity of cerebellar microcircuits in vermal lobule V-VI, which are modified at the level of synaptic plasticity and affect fear learning and memory, influences neural activity in the DCN or DCN-targeting regions. Investigating the cerebellar efferent pathways involved in fear learning and memory would also be interesting since it is plausible that the distinct cerebellar regions may contribute to different components or phases of fear memory *via* distinct connectivity patterns. Several studies targeting the vermis containing mainly lobule V-VI found that the lobule V-VI is involved in fear consolidation or retrieval (Sacchetti et al., 2002; Dubois et al., 2020; Han et al., 2021). On the other side, the lobule VIII regulates vlPAG activation-induced muscle tone inducing freezing behavior (Koutsikou et al., 2014). In DCN, the IpN is involved in fear consolidation, whereas the FN projecting to vlPAG is involved in fear acquisition and extinction but not consolidation (Sacchetti et al., 2002; Frontera et al., 2020). Further studies investigating the connections between the cerebellar cortex and the DCN as well as between the DCN subnuclei and extra-cerebellar regions can provide an integrated understanding of the cerebellar roles in fear learning and memory. In addition, for a more detailed understanding of cerebellar fear processing and other non-motor functions, future studies should consider cerebellar molecular patterns, including zebrin expression patterns and molecular heterogeneity.

Then, what is the role of the cerebellum in the fear network? As mentioned above, inactivating the amygdala alone was insufficient to block the retrieval of auditory fear when mice were trained with a US of a higher intensity, which induced a strong fear memory (Sacchetti et al., 2007). Strong fear memories were suppressed by combined inactivation of the amygdala and cerebellum, suggesting that the cerebellum is critical for processing strong fear memories (Sacchetti et al., 2007). In addition, cerebellar lesions or dysregulation seem to have a greater and more general impact on cued fear memory than on contextual fear memory (Sacchetti et al., 2002; Dubois et al., 2020; Han et al., 2021). As the cerebellum encodes sensory prediction error and timing, thereby contributing to motor coordination as well as associative learning paradigms including reward learning and eyeblink conditioning, it is likely that the cerebellum enables animals to make appropriate responses to sensory cues associated with aversive stimuli (Ten Brinke et al., 2017; Heffley and Hull, 2019). Furthermore, the findings that the unexpected omission of US activates the cerebellum and that inhibition of FN-vlPAG during extinction learning impairs fear extinction learning strongly support that the cerebellum is a necessary locus for processing prediction errors that violate the expectation of the US (Ernst et al., 2019; Frontera et al., 2020). Thus, the cerebellum may contribute to cued fear learning processes by adjusting its level of prediction error (Bouton, 2004; Ernst et al., 2019; Frontera et al., 2020).

Considering its role in sensory processing and prediction, the cerebellum is highly likely to be involved in many, if not all, types of cued learning as far as discrete sensory stimuli are used as CSs (Ten Brinke et al., 2017; Heffley and Hull, 2019). One of the common characteristics between fear conditioning and other associative learning paradigms is the association of a neutral and salient sensory stimulus either with an aversive unconditioned stimulus or with a reward (Myers and Davis, 2007; Tovote et al., 2015; Ten Brinke et al., 2017; Heffley and Hull, 2019). In addition to the eyeblink conditioning which is the most well-studied cerebellum-dependent cued learning, recent studies show that reward-based operant learning paradigms such as go/no-go test which involves sensory cues also requires the cerebellum (Wagner et al., 2017; Heffley et al., 2018). Nevertheless, a cerebellar role in contextual fear learning and memory cannot be overlooked as cerebellar manipulations targeting the cerebellar vermis and hemispheres affect contextual fear conditioning (Supple et al., 1988; Sacchetti et al., 2002). Further studies focusing on the lateral cerebellum may shed light on the cerebellar role in contextual fear conditioning as the lateral cerebellum is thought to engage more cognitive functions than the medial cerebellum (Supple et al., 1988; D’mello et al., 2020).

Cerebellar dysfunction is associated with motor diseases as well as cognitive and affective disorders, including post-traumatic stress disorder, autism spectrum disorders, and depressive-like behavior (De Bellis and Kuchibhatla, 2006; Tsai et al., 2012; Rabellino et al., 2018; Kelly et al., 2020; Baek et al., 2022). Identifying and understanding the reciprocal communication between the cerebellum and other brain regions will be critical for elucidating the pathophysiology underlying cerebellum-associated non-motor brain disorders. Indeed, recent studies have begun to identify cerebellar output target regions that modulate non-motor cognitive and affective behaviors (Carta et al., 2019; Baek et al., 2022). In conclusion, understanding the cerebellar circuits underlying fear learning and memory and the accompanying plasticity may contribute to the development of novel treatment strategies for affective disorders.

## Author Contributions

K-DH, SK, and Y-SL reviewed the literature and wrote the manuscript. All authors contributed to the article and approved the submitted version.

## Conflict of Interest

The authors declare that the research was conducted in the absence of any commercial or financial relationships that could be construed as a potential conflict of interest.

## Publisher’s Note

All claims expressed in this article are solely those of the authors and do not necessarily represent those of their affiliated organizations, or those of the publisher, the editors and the reviewers. Any product that may be evaluated in this article, or claim that may be made by its manufacturer, is not guaranteed or endorsed by the publisher.
